# Dangers of Hypnotism

**Published:** 1890-11

**Authors:** 


### Dangers of Hypnotism.

It is a lamentable fact that there is to be found in the dental ranks some who are advocates of hypnotism as a means toward alleviating pain during dental operations. It is, however, a pleasure to state that the number is few. Happily it is but few can master the details in the performance of hypnotism. All the better class of dentists would not practice it even if they could, knowing that such a practice would lead patients and their friends to an abhorrence of the practitioner for fear that some undue advantage would be taken of them.

The following taken from the *Medical Record*, will be of interest to every student of this subject:

"At the late meeting of the British Medical Association Dr. Norman Kerr, of London, introduced the subject of hypnotism. He said that he accepted practically all the alleged hypnotic phenomena as facts. But in hypnosis, after close watching, he saw only a distorted cerebral state, an abnormal physical condition with exaltation of receptivity and energy. Was hypnotism a desirable and justifiable remedy? Several considerations must be taken into account in answering this question. 1. Only a limited number of persons were susceptible. 2 The after-effect was a disturbance of mental balance, a dissipation of nerve energy and nerve exhaustion. Frequent repetition was apt to cause deterioration of brain and nerve function, intellectual decadence, and moral perversion. 3. Hypnosis was a departure from health; a diseased state. 4. Hypnosis was a true neurosis embracing the lethargic, cataleptic, and somnambulistic states. Thus, if a disease were cured by hypnotism this would be only by substituting another disease. The suffering was sometimes temporarily assuaged by hypnotic suggestion, but the underlying disease was not necessarily cured by hypnotic anæsthesia though evanescent oblivion might be secured. 5. The lethel power of the morbid disorder, of which the pain was a merciful, if unwelcome messenger, was in most cases increased. The few cases he had seen apparently benefited would probably have yielded to ordinary treatment, but the patients resisted or were passive to that, while they looked forward to, believed in, and gave themselves up to the mesmerizer. 6. The dangers of hypnotism were very great. Each seance might bring the hypnotee more under the control of the hypnotist, ending often in the complete submission of the former to the will of the latter. A jelly-fish slavery without mental or moral backbone, was infinitely worse than days of pain and nights of agony. There were many wrecked lives through mesmerism. 7. An elective and subtle activity, ending in disaster, might develop between operator and operated upon. 8. In the lethargic and cataleptic states criminal assaults had been committed by medical men who had been convicted and punished.

"In the somnambulistic state subjects had been compelled by the operator's behest to commit crime. So serious were these evils that French surgeons had been prohibited from practicing hypnotism in the army and navy. 9. It is not desirable that the control of any one's thought and action should be in the keeping of a fallible fellow-mortal."

In view of all these possible dangers Dr. Kerr could not understand why medical men in family practice should have been incited to hypnotize patients of both sexes and all ages in the daily round of domiciliary visiting. He strongly deprecated public mesmerism—medical, philanthropic, or commercial—as degrading and disgusting, and particularly centured the medical patronage and indorsement thereof.

The REGISTER is ever ready to give place to all articles that tend to elevate the dental profession, and with the reading of this quotation we hope that it will inaugurate within each and every one a pledge to discountenance such unprofessional and unprincipled practice. W. H. W.

